# Regulator of chromatin condensation 1 abrogates the G1 cell cycle checkpoint via Cdk1 in human papillomavirus E7-expressing epithelium and cervical cancer cells

**DOI:** 10.1038/s41419-018-0584-z

**Published:** 2018-05-22

**Authors:** Lijun Qiao, Jingyi Zheng, Yonghao Tian, Qishu Zhang, Xiao Wang, Jason J. Chen, Weifang Zhang

**Affiliations:** 1 Cancer Research Center and Department of Microbiology, School of Basic Medical Sciences, Shandong University, Jinan, Shandong China; 2 Department of Microbiology and Key Laboratory of Infection and Immunity of Shandong Province, School of Basic Medical Sciences, Shandong University, Jinan, Shandong China; 3 Department of Orthopedic Surgery, Qilu Hospital Affiliated Shandong University, Jinan, Shandong China; 4 Institute of Pathobiology, School of Basic Medical Sciences, Shandong University, Jinan, Shandong China

## Abstract

Regulator of chromatin condensation 1 (RCC1) is a major guanine-nucleotide exchange factor for Ran GTPase and plays key roles in nucleo-cytoplasmic transport, mitosis, and nuclear envelope assembly. RCC1 is known to be a critical cell cycle regulator whose loss causes G1 phase arrest, but the molecular basis for this regulation is poorly understood. Furthermore, little is known about the relationship between RCC1 and carcinomas. Human papillomavirus (HPV) infection is highly associated with the development of cervical cancer. The expression and function of RCC1 in HPV-related cervical cancer and cell cycle regulation have not yet been explored. In this study, we first observed that RCC1 immunostaining was mildly increased in cervical cancer tissues and significantly upregulated in HPV E7-expressing cells; this localization was primarily nuclear. We showed that the transcription factor c-Jun transcriptionally upregulates RCC1 via a direct interaction with the RCC1 promoter. Moreover, siRNA-mediated knockdown of RCC1 inhibited G1/S cell cycle progression and DNA synthesis, while overexpression of RCC1 abrogated the G1 checkpoint. RCC1 knockdown downregulated the protein levels of the transcription factor E2F1, especially nuclear E2F1, by promoting its degradation in HPV E7-expressing cells. Overexpression of E2F1 rescued RCC1 knockdown-mediated inhibition of G1/S progression. Additionally, we showed that cyclin-dependent kinase 1 (Cdk1), a known target of E2F1, is involved in G1 checkpoint regulation, as Cdk1 knockdown hindered G1/S progression, while Cdk1 overexpression rescued RCC1 knockdown-mediated effect on G1 cell cycle progression. Furthermore, RCC1 knockdown reduced HPV E7 protein levels, which may in turn downregulate E2F1. Our study explores the function of RCC1 in G1/S cell cycle progression and suggests that RCC1 may be involved in HPV E7-mediated genomic instability.

## Introduction

Cervical cancer is one of the most common malignancies in females worldwide^1^ and is commonly associated with high-risk human papillomavirus (HR-HPV) infection^2,3^. HPVs are small DNA viruses that replicate in squamous epithelium. The HPV oncogenic proteins E6 and E7 bind to and degrade tumor suppressor p53 and retinoblastoma (pRb), respectively, thus regulating many key cellular processes such as proliferation and transformation^4,5^. High-risk HPV (such as HPV-16, 18 etc.), E7 protein, which is consistently expressed in cervical cancer and possesses the major transforming activity, abrogates cell cycle checkpoints and induces genomic instability^6^. Although numerous E7 interacting proteins have been identified, there are still many unknown proteins that may be involved in E7-mediated cell cycle regulation and transformation.

RCC1 (regulator of chromatin condensation 1) was first identified during premature chromosomal condensation in BHK cells^7^. In recent years, studies have shown that RCC1 is a guanine-nucleotide exchange factor (GEF) that acts on the nuclear Ras-like small GTPase Ran^8^. RCC1 has been shown to be a critical cell cycle regulator and a component of a GTPase switch that monitors the progress of DNA synthesis and couples the completion of DNA synthesis to the onset of mitosis^9–12^. RCC1 is further involved in nucleo-cytoplasmic transport, mitotic spindle formation, and nuclear envelope assembly following mitosis^13,14^. Increased RCC1 expression could raise cellular RanGTP levels and enhance the function of importin β and exportin 1, which accelerate cell cycle progression and modulate cellular responses to DNA damage^15^. Loss of RCC1 might block cell cycle progression though the G1/S transition^14^. Although the role of RCC1 in mitosis has been well documented, the molecular basis of RCC1-mediated G1/S transition is far from completely understood.

The role of RCC1 in carcinoma is uncertain. RCC1 was identified as being overexpressed in mantle-cell lymphoma^16^. Another report showed that RCC1 expression was significantly higher in lung adenocarcinoma tissues compared with adjacent normal tissues^17^. These results suggest that RCC1 may promote cancer formation. Proteomic profiling revealed that RCC1 was decreased in HepG2 hepatoma cells induced with 6-bromine-5-hydroxy-4-methoxybenzaldehyde^18^. Another report demonstrated that RCC1 expression was significantly lower in gastric carcinoma tissues and that methylation-induced silencing of RCC1 expression was associated with tumorigenesis and depth of invasion in gastric cancer, suggesting that RCC1 may be a tumor suppressor in gastric carcinoma^19^. Genome-wide transcriptional analysis of the carboplatin response in chemo-sensitive and chemo-resistant ovarian cancer cells indicated that RCC1 expression was higher in carboplatin-sensitive cells^20^. However, in colorectal carcinoma cells, RCC1 was reported to promote doxorubicin resistance^15^. All of these data indicate that differences in RCC1 expression and function may depend on the type of tumor. Importantly, whole genome expression profiling of progressive stages of cervical cancer indicated that RCC1 was overexpressed in International Federation of Gynaecology and Obstetrics (FIGO) Stage III cervical cancer tissues compared to normal cervix^21^. However, the role of RCC1 in cervical cancer and in HPV E7-expressing cells is largely unknown.

Data from GEO datasets showed that RCC1 was overexpressed in cervical cancer as well as HPV-related cervical cancer. Furthermore, immunostaining demonstrated that RCC1 protein was slightly increased in cervical cancer tissues compared with normal cervix. HPV E7 markedly upregulated RCC1 expression via c-Jun. Furthermore, knockdown of RCC1 reduced E7-induced G1 checkpoint abrogation. In this study, we show that RCC1 mediates G1 cell cycle progression in an E2F1-dependent manner and that overexpression of E2F1 rescues the RCC1 knockdown-mediated inhibition of the G1/S transition. We also demonstrate a vital role for Cdk1 in G1 checkpoint abrogation. These results may provide insights into the cellular pathways targeted during HPV-induced carcinogenesis.

## Results

### High RCC1 expression correlates with cervical cancer and HPV-related cervical cancer

**Fig. 1:**
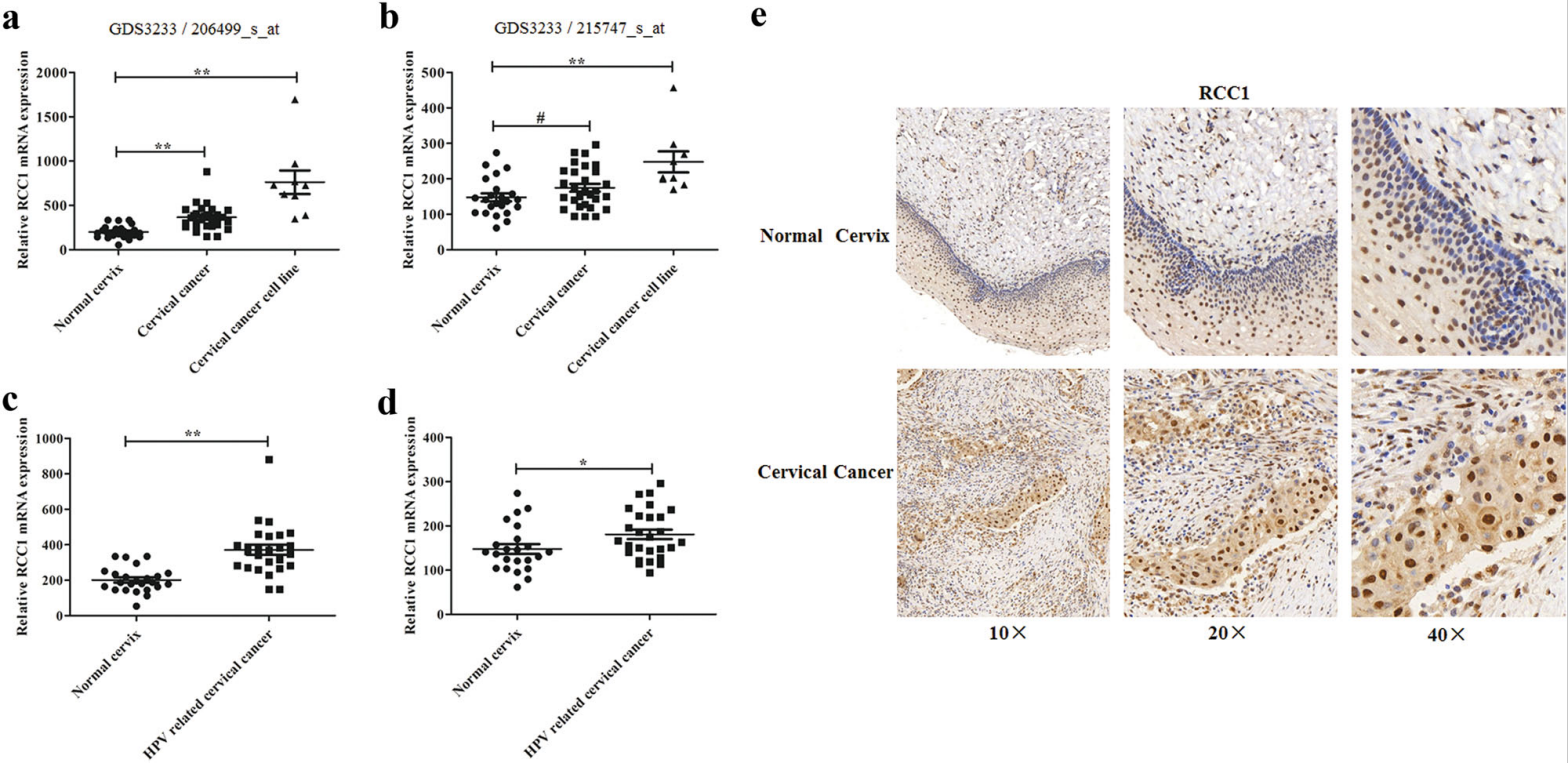
High RCC1 expression correlates with cervical cancer and HPV-related cervical cancer. RCC1 gene expression profiles from cervical cancer samples were downloaded from the NCBI GEO database (accession No. GDS3233), including 24 normal cervical epithelium, 33 primary cancers and nine cell lines. **a**, **b** RCC1 mRNA expression (probe set 206499_s_at, 215747_s_at) in cervical cancer. **c**, **d** RCC1 mRNA expression (probe set 206499_s_at, 215747_s_at) in HPV-related cervical cancer. **e** Representative results of immunohistochemical staining for RCC1 expression in cervical cancer tissues and normal cervix tissues. ^#^*P* < 0.1; *P < 0.05; ** *P* < 0.01

Whole genome expression profiling of progressive stages of cervical cancer indicated that high RCC1 expression was closely correlated with FIGO Stage III^21^. To further confirm the relationship between RCC1 expression and cervical cancer, raw cervical cancer microarray data were downloaded from the NCBI GEO database (accession No. GDS3233). A total of 66 samples were included in this study, including 24 normal cervical epitheliums, 33 primary cancers, and nine cell lines^22^. We found that RCC1 mRNA expression (the probe set 206499_s_at, 215747_s_at) was upregulated in cervical cancer tissues and cell lines, including SiHa and HeLa, in comparison with normal cervical epithelium (Fig. 1a,b). Since human papillomavirus (HPV) is the main etiologic factor in cervical cancer, we speculated that RCC1 expression was correlated with HPV infection. Among the 33 primary cancers, there were only two HPV-negative samples. Eliminating these two HPV-negative samples, RCC1 expression was still significantly higher in HPV-positive cervical cancers (Fig. 1c,d). These results suggest that RCC1 may be involved in cervical carcinogenesis.

To confirm these observations, we performed immunohistochemical staining for RCC1 protein in normal cervix (*n* = 11 samples) and cervical cancer tissues (*n* = 47 samples). We detected a weak cytoplasmic signal for RCC1 protein in all normal cervix and cervical cancer tissues. Nuclear RCC1 expression was mildly increased in cervical cancer tissues, but there was no significant difference in staining intensity scores between cervical cancer and normal cervix tissues (*P* < 0.50) (Fig. 1e).

### RCC1 expression is upregulated by c-Jun in HPV E7-expressing cells

**Fig. 2:**
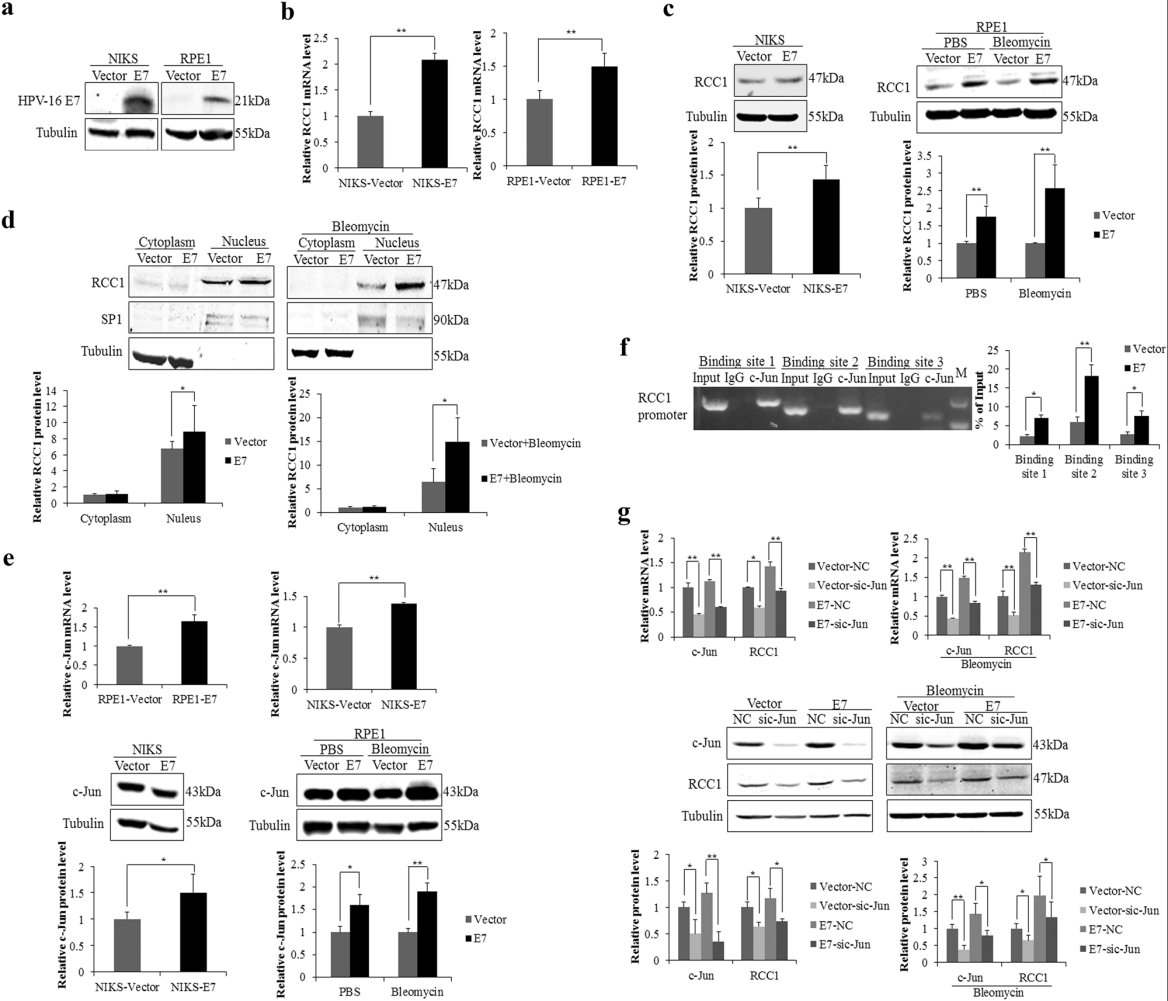
RCC1 expression was upregulated by c-Jun in HPV E7-expressing cells. **a** Expression of HPV-16 E7 in NIKS and RPE1 cells was confirmed by Western blot. **b** RCC1 mRNA levels in NIKS and RPE1 cells were determined by real-time RT-PCR. **c** Steady-state RCC1 protein levels in NIKS and RPE1 cells were determined by Western blot. **d** Cellular localization of RCC1 in RPE1 cells and bleomycin (10 μg/mL) treated RPE1 cells. Lower panel, quantification of RCC1 protein expression in different cellular compartments. **e** The steady-state c-Jun mRNA and protein levels in NIKS and RPE1 cells, as determined by real-time RT-PCR and Western blot. **f** ChIP assays were performed using a c-Jun antibody in cultured RPE1-E7 and RPE1-Vector cells. The c-Jun-associated RCC1 promoter binding sites 1, 2 and 3 were detected by PCR with RCC1 promoter primers. Input, total DNA. **g** RCC1 mRNA and protein levels were measured by real-time RT-PCR and Western blot 24 and 48 h after transfection with siRNAs targeting c-Jun. Data representative of three biological replicates are shown. Data are presented as the means and standard deviations (SD). *P < 0.05; **P < 0.01

The HPV E7 oncoprotein possesses the major transforming activity in cervical carcinogenesis. To confirm the regulation of RCC1 by HPV E7, we first used HPV-16 E7-expressing NIKS cells (NIKS-E7)^23^. NIKS cells are natural host cells for HPV and are able to maintain the HPV life cycle^24,25^. Before we conducted experiments, we confirmed the expression of HPV-16 E7 (Fig. 2a). We showed that RCC1 mRNA levels were upregulated (~2-fold) in E7-expressing NIKS cells (Fig. 2b). It is very difficult to achieve a high transfection efficiency with keratinocytes, however, RPE1 cells are much easier to transfect and possess intermediate mitotic RanGTP levels^26^. Thus, we used RPE1 cells expressing the vector control (RPE1-Vector) or wild-type HPV-16 E7 (RPE1-E7) for our HPV-related functional studies^27–29^. Similar to observations made in keratinocytes, RCC1 mRNA levels were increased (~1.5-fold) in E7-expressing RPE1 cells (Fig. 2b). We further examined the steady-state RCC1 protein levels. As shown in Figure 2c, the levels of RCC1 protein were significantly upregulated in both NIKS-E7 cells (~1.5-fold) and RPE1-E7 cells (~1.7-fold). Following activation of the G1 checkpoint with the DNA-damaging agent bleomycin, we observed that RCC1 protein levels were still higher in bleomycin-treated RPE1-E7 cells (Fig. 2c).

RCC1 dynamically associates with chromatin through its N-terminal tail, which is capable of binding to histones H2A and/or H2B in a Ran-regulated manner^30^. We next examined the cellular localization of RCC1 protein. Our results showed that the RCC1 protein was mainly located in the nucleus, where its level was approximately 1.3-fold higher in E7-expressing cells than in control RPE1 cells (Fig. 2d, left panel). When treated with bleomycin, nuclear RCC1 protein levels were approximately 2-fold higher in E7-expressing RPE1 cells (Fig. 2d, right panel).

c-Jun, which functions as a homodimer with itself or a heterodimer with other activator protein-1 (AP-1) family members, is a key transcription factor^31^. c-Jun recognizes and binds to the heptamer enhancer motif 5'-TGAC/GTCA-3'^32^. c-Jun has important roles in many cellular processes, such as proliferation, transformation, and apoptosis^31,33,34^. In our study, both c-Jun mRNA and protein levels were upregulated in HPV E7-expressing NIKS and RPE1 cells as well as bleomycin-treated RPE1-E7 cells (Fig. 2e). Several TGAC/GTCA c-Jun binding sites can be found in the promoter of RCC1, so we speculated that c-Jun may regulate the expression of RCC1. To test this possibility, we performed chromatin immunoprecipitation (ChIP) assays to examine the loading of c-Jun onto chromatin. As shown in Figure 2f, c-Jun bound to chromatin at three sites within the RCC1 promoter and significantly more c-Jun bound to chromatin in E7-expressing cells than in vector control cells. To further verify the regulation of RCC1 by c-Jun, we transfected cells with siRNAs specific to c-Jun. Steady-state c-Jun protein levels were downregulated (to 0.3-fold) following siRNA transfection. Furthermore, RCC1 mRNA and protein levels were significantly downregulated following knockdown of c-Jun (Fig. 2g). These results suggest that RCC1 might be regulated by c-Jun at the transcriptional level.

### RCC1 knockdown inhibits G1/S progression and DNA replication

**Fig. 3:**
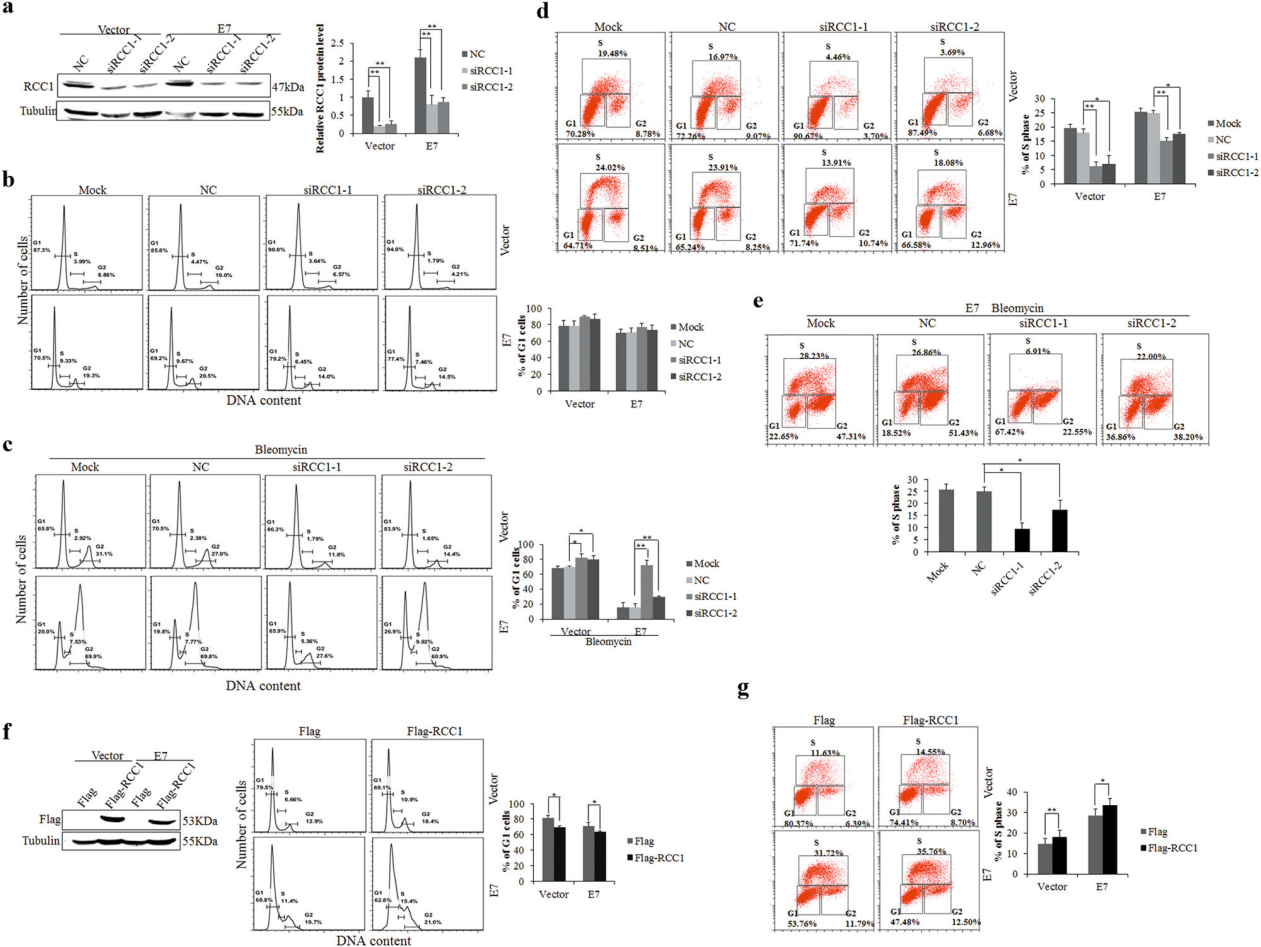
RCC1 knockdown inhibits G1/S progression and DNA replication. **a** RCC1 protein levels were measured by Western blot 48 h after transfection with siRNAs targeting RCC1. **b**, **c** Flow cytometry of cells transfected with siRCC1 for 24 h, treated with or without bleomycin for 36 h, then stained with PI. G1, S, and G2 phases are indicated. **d** Flow cytometry of cells transfected with RCC1 siRNAs for 48 h and labeled with 20 nM BrdU for 2 additional hours. Cells were stained with an anti-BrdU antibody, counterstained with 7-AAD, and analyzed by flow cytometry. **e** Flow cytometry of E7-expressing RPE1 cells transfected with RCC1 siRNAs for 24 h and treated with bleomycin for 36 h. **f** RCC1 protein levels and cell cycle distribution were measured after transfection with a plasmid encoding RCC1. Flow cytometry of cells transfected with a plasmid encoding RCC1 for 48 h, then stained with PI. **g** Flow cytometry of cells transfected with a plasmid encoding RCC1 for 48 h and labeled with 20 nM BrdU for 2 additional hours. Data representative of 3 biological replicates are shown. *P < 0.05; **P < 0.01

RCC1 was reported to regulate the G1/S cell cycle transition^9,35^, but the underlying mechanisms have not been fully studied. To explore the role of RCC1, we used siRNAs specific to RCC1. The steady-state levels of RCC1 protein in E7-expressing cells were downregulated by approximately 50% after transfection with siRCC1s, which was close to the protein levels of RCC1 in vector control cells (Fig. 3a). Next, we examined the ability of RCC1 to modulate the G1 checkpoint in E7-expressing RPE1 cells. Under normal conditions, although the average G1 phase percentage increased with RCC1 knockdown, there were no significant differences (Fig. 3b). To activate the G1 checkpoint, we treated cells with bleomycin (10 μg/mL), which causes both single and double strand DNA breaks and induces G1 arrest^28,36^. Consistent with what we have recently observed, upon treatment with bleomycin, fewer E7-expressing cells than vector control cells (20.0% vs 65.8%) arrested in G1 (Fig. 3c, the first column), indicating that HPV E7 abrogated the G1 checkpoint. Notably, knockdown of RCC1 led to a significant increase in the G1 peak in both E7-expressing and control RPE1 cells (Fig. 3c). Furthermore, RCC1 knockdown increased more number of E7-expressing cells arrested in G1 than control RPE1 cells. To demonstrate the role of RCC1 in promoting S phase entry, we transfected siRNAs targeting RCC1 into RPE1 cells and measured BrdU incorporation. Knockdown of RCC1 led to a significant reduction in BrdU incorporation in both RPE1-E7 cells and RPE1-Vector cells (Fig. 3d). When treated with bleomycin, we observed a marked decrease in BrdU incorporation in siRCC1-treated RPE1-E7 cells (Fig. 3e). To further verify the role of RCC1 in G1, cells were transfected with a plasmid encoding RCC1. As expected, overexpression of RCC1 accelerated cell cycle progression by promoting the G1/S transition, resulting in fewer G1 phase cells and greater S phase cells in both PBS and bleomycin-treated cells (Fig. 3f,g). These results suggest an important role for RCC1 in G1 cell cycle control and S phase entry.

### RCC1 knockdown promotes E2F1 degradation and E2F1 overexpression rescues the RCC1 knockdown-mediated inhibition of the G1/S transition

**Fig. 4:**
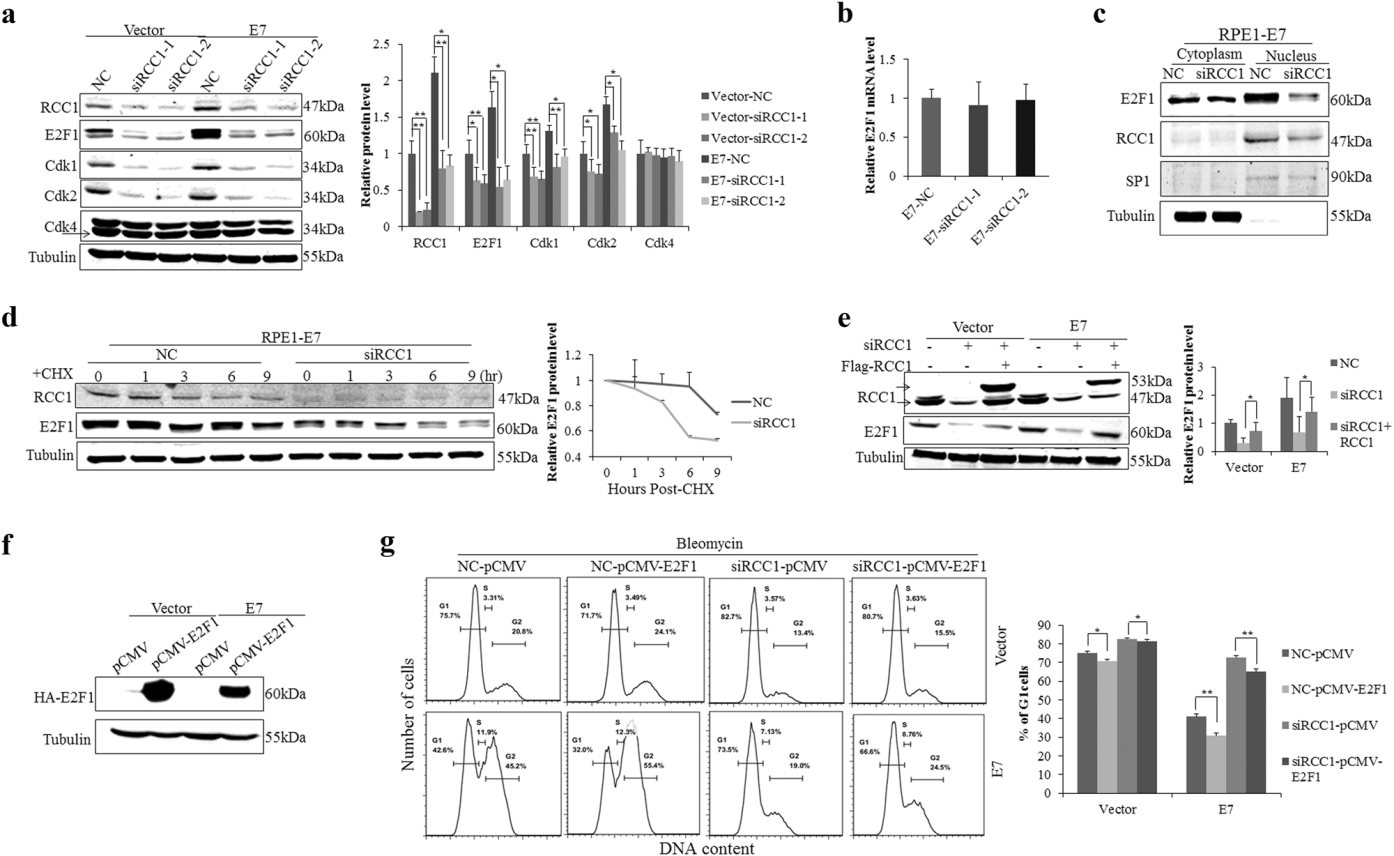
RCC1 knockdown promotes degradation of E2F1 and E2F1 overexpression rescues the RCC1 knockdown-mediated inhibition of the G1/S transition. **a** Western blot analysis of RCC1, E2F1, Cdk1, Cdk2, and Cdk4 following RCC1 knockdown. **b** Real-time RT-PCR analysis of E2F1 with RCC1 knockdown. **c** Western blot analysis of E2F1 cellular localization in E7-expressing cells following RCC1 knockdown. **d** RPE1-E7 cells were incubated with 25 μg/mL cycloheximide (CHX) and harvested at the indicated times. E2F1 stability was monitored by Western blot. E2F1 half-life was measured and calculated using the Half Life Calculator. **e** RCC1 and E2F1 protein levels were measured after siRNA-mediated knockdown of endogenous RCC1 and transfection with a mutant plasmid resistant to siRCC1. **f** Western blot analysis of HA-E2F1 following transfection of cells with pCMV or pCMV-E2F1 plasmid. **g** Flow cytometry of cells transfected with RCC1 siRNAs and either pCMV or pCMV-E2F1 plasmid for 24 h and then treated with bleomycin for 36 h. Data representative of three biological replicates are shown. *P < 0.05; **P < 0.01

To explore how RCC1 regulates the G1/S transition, we detected the expression of genes involved in the G1 checkpoint: E2F1, Cdk1, Cdk2, and Cdk4. E2F1, Cdk1, and Cdk2 protein levels were considerably higher in E7-expressing cells and were markedly decreased upon RCC1 knockdown, however, no significant change in Cdk4 protein levels was observed (Fig. 4a). These results suggest a role for E2F1, Cdk1, and Cdk2 in RCC1-mediated G1/S transition in E7-expressing cells.

E2F1 is generally considered to be ‘active E2F’, as its binding to promoters results in increased transcription^37^. E2F1 expression was higher in E7-expressing cells and the steady-state E2F1 level was downregulated upon RCC1 knockdown (Fig. 4a). To explore how RCC1 affects E2F1, we conducted real-time RT-PCR. E2F1 mRNA levels stayed relatively constant after RCC1 knockdown, which suggests that RCC1 does not affect E2F1 at the transcriptional level (Fig. 4b). Next, we performed cell fractionation assays. As shown in Figure 4c, RCC1 knockdown (siRCC1-1) resulted in a decrease in nuclear E2F1 but did not seem to affect E2F1 levels in the cytoplasm. We speculate that knockdown of RCC1 may lead to the degradation of E2F1. To test this idea, we detected E2F1 protein stability after treatment with cycloheximide (CHX), which inhibits protein synthesis. As shown in Figure 4d, the steady-state E2F1 levels dropped more quickly with RCC1 knockdown, indicating that E2F1 degradation was increased. Then, endogenous RCC1 was knocked down using siRNAs and cells were transfected with a Flag-RCC1 plasmid encoding a knockdown-resistant RCC1 protein harboring an altered siRCC1-1 target sequence. As shown in Figure 4e, E2F1 protein levels recovered when cells were transfected with Flag-RCC1 (the lower band represents endogenous RCC1, while the upper band represents exogenous RCC1), indicating that RCC1 protected the stability of E2F1. To further verify that RCC1 regulates E2F1 activity during G1/S cell cycle progression, cells were transfected with a plasmid encoding E2F1. The expression of HA-tagged E2F1 was confirmed (Fig. 4f). Overexpression of E2F1 induced cells to further bypass G1 arrest (Fig. 4g). Knockdown of RCC1 caused an accumulation of cells in G1 (73.5% vs 42.6%) while overexpression of E2F1 partly bypassed G1 arrest (66.6% vs 73.5%) in E7-expressing cells (Fig. 4g, the second row). Thus, E2F1 overexpression rescued the RCC1 knockdown-induced inhibition of the G1/S transition. These results suggest that RCC1 might regulate G1/S cell cycle progression in an E2F1-dependent manner.

### Cdk1 abrogates the G1 checkpoint

**Fig. 5:**
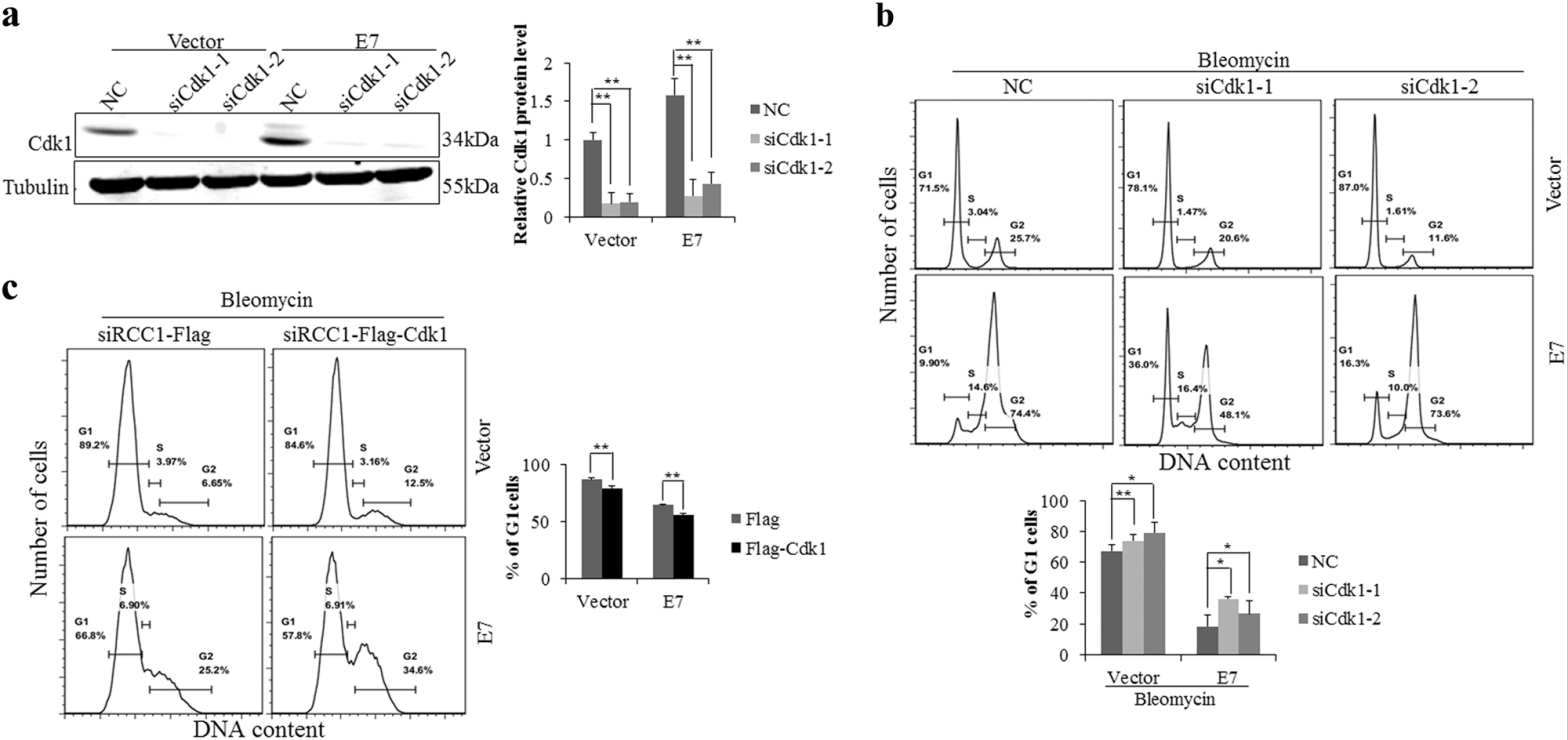
Cdk1 induces G1 checkpoint abrogation. **a** Cdk1 protein levels were measured by Western blot 48 h after transfection with siRNAs targeting Cdk1. **b** Flow cytometry of cells transfected with Cdk1 siRNAs for 24 h, treated with bleomycin for 36 h, then stained with PI. **c** Flow cytometry of cells transfected with siRCC1 and Flag or Flag-Cdk1 for 24 h and then treated with bleomycin for 36 h. Data representative of three biological replicates are shown. *P < 0.05; **P < 0.01

Cell cycle progression is regulated by Cdks and cyclins at several checkpoints. Cdk2 has been considered the master kinase for S phase entry^38^, but Cdk1 can functionally compensate for Cdk2 in the absence of this kinase^39^. Accumulating evidence indicates that Cdk1 plays a part in G1/S phase transition^36^. As shown in Figure 5a, specific Cdk1 knockdown was achieved in RPE1 cells. Significantly, transfection of siRNAs targeting Cdk1 led to more increase in the number of G1 phase bleomycin-treated E7-expressing cells (Fig. 5b). To further confirm the role of Cdk1 in the G1 checkpoint, we overexpressed Cdk1 protein. With overexpression of Cdk1, cells with RCC1 knockdown partially bypassed G1 arrest (Fig. 5c). Therefore, Cdk1 indeed rescues the effect of RCC1 knockdown on G1 arrest.

### RCC1 knockdown induces G1 arrest in cervical cancer cells

**Fig. 6:**
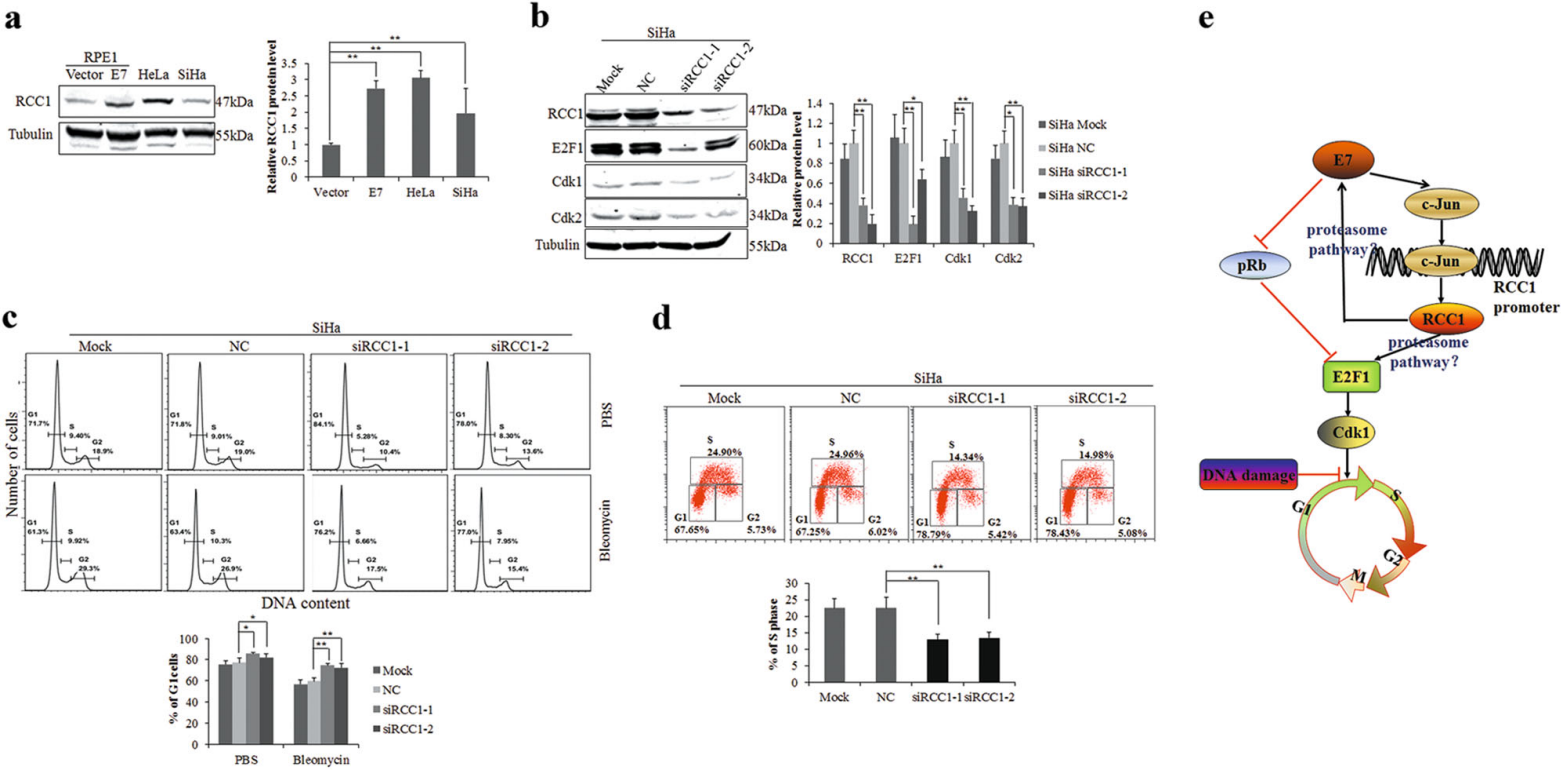
RCC1 knockdown induces G1 arrest in cervical cancer cells. **a** RCC1 protein levels were measured by Western blot in HeLa and SiHa cells. **b** Western blot analysis of E2F1, Cdk1, and Cdk2 protein levels in SiHa cells transfected with RCC1 siRNAs. **c** Flow cytometry of SiHa cells transfected with RCC1 siRNAs for 24 h and treated with bleomycin for 36 h. **d** Flow cytometry of SiHa cells transfected with RCC1 siRNAs for 48 h, then stained with anti-BrdU and 7-AAD. **e** A model depicting the E7/c-Jun/RCC1 pathway. In this model, E7 binds to and degrades Rb and releases E2F1 from the pRb-E2F1 complex. E7 also upregulates c-Jun, allowing c-Jun to bind to the RCC1 promoter and increase its transcriptional activation. Increased RCC1 upregulates the steady-state levels of E2F1, likely through the proteasome pathway. Moreover, RCC1 positively regulates the steady-state levels of E7 oncoprotein, which in turn promotes E2F1. Cdk1, a target of E2F1, plays a role in G1/S progression in the presence of Cdk2. This E7/c-Jun/RCC1/E2F1/Cdk1 pathway may lead to abrogation of the G1 checkpoint in the presence of damaged DNA, resulting in dysregulation of the G1/S cell cycle. Data representative of three biological replicates are shown. *P < 0.05; **P < 0.01

As shown in the NCBI GEO dataset (accession No. GDS3233), RCC1 mRNA levels were higher in cervical cancer cells, including HeLa and SiHa. We showed that the steady-state RCC1 protein levels were higher in HeLa and SiHa cells compared with RPE1-Vector cells (Fig. 6a).

To confirm the function of RCC1 in the cell cycle, we treated SiHa cervical cancer cells with siRCC1s and observed downregulation of E2F1, Cdk1, and Cdk2 protein levels (Fig. 6b). As we expected, knockdown of RCC1 led to an increase in the G1 peak upon treatment with bleomycin (Fig. 6c) and a significant reduction in BrdU incorporation (Fig. 6d). Similar results were observed in HeLa cervical cancer cells (Supplementary Figure S1). These results demonstrate an important role for RCC1 in G1/S cell cycle control in cervical cancer cells.

## Discussion

In this study, we explored the cell cycle-promoting activity of RCC1 in HPV E7-expressing epithelium and cervical cancer cells. We first showed that RCC1 was upregulated by c-Jun in both cervical cancer tissues and HPV-16 E7-expressing cells. Next, we showed that RCC1 was involved in E7-mediated abrogation of the G1 checkpoint through regulation of E2F1 degradation. We further showed that Cdk1, an E2F1 target, can rescue G1/S progression rates. Taken together, this study is the first to identify a relationship between RCC1 and cervical cancer and uncovers a novel function of RCC1 in high-risk HPV E7-mediated G1/S cell cycle control. These finding have important implications for HPV-related cancers.

c-Jun, an AP-1 family member, is a key transcription factor that forms homo-dimers or hetero-dimers with members of the Fos and ATF families^31^. c-Jun/AP-1 may be activated by various factors, such as growth factors, environmental stresses, and hormones. ERK1/2, a mitogen-activated protein kinase (MAPK), has been reported to be overexpressed in cervical cancer tissue and to promote cervical cancer cell growth by regulating the expression of c-Jun and c-fos^40^. This indicates that activation of c-Jun through the MAPK pathways may lead to tumor progression. In our study, c-Jun was upregulated in E7-expressing cells and c-Jun was able to bind to the promoter of RCC1 to activate the expression of RCC1, thereby accelerating cell cycle progression.

Because RCC1 has important roles in nucleo-cytoplasmic transport, lack of RCC1 can cause nucleo-cytoplasmic transport collapse^41^, reducing the levels of mRNAs required for DNA replication and nuclear export and shutting off synthesis of the proteins required for the G1/S transition. However, this is not a reasonable explanation for the inability of cells to bypass the G1/S transition in our system, as we knocked down RCC1 protein levels close to levels in vector control cells to make sure the normal physiological activity of cells was not affected. Thus, the E2F1 and Cdk1 downregulation following RCC1 knockdown cannot be simply attributed to the effect of altered nucleo-cytoplasmic transport.

E2F1 was found to bind to upstream regions within the human Cdk1 and Cdk2 genes and to accelerate the transcription of these genes^42,43^. Our data proved that E2F1 upregulates Cdk1 and Cdk2 protein levels (not shown). We noticed that both RCC1 and E2F1 were upregulated by HPV E7. Meanwhile, E2F1 protein levels were downregulated following RCC1 knockdown in E7-expressing cells. These results demonstrate a positive correlation between RCC1 and E2F1 and propose that RCC1 might control the G1/S transition by regulating the Cdks in an E2F1-dependent manner. Although E2F1 expression decreased following RCC1 knockdown, the mechanism by which RCC1 regulates E2F1 expression remains to be determined. In the present study, E2F1 protein levels were markedly decreased in the nucleus following RCC1 knockdown. Furthermore, E2F1 protein levels were reduced more rapidly when protein synthesis was inhibited by CHX, suggesting that RCC1 knockdown may promote the degradation of E2F1. However, the underlying molecular mechanism needs to be further studied. E2F1 is mainly ubiquitinated and degraded by the ubiquitin-protein ligase Skp2^44^. A series of studies have demonstrated that E2F1 post-translational modifications are related to E2F1 subcellular localization and can lead to degradation via the ubiquitin–proteasome pathway^45,46^. p38 MAPK phosphorylates E2F1 on residues Ser403 and Thr433, which results in E2F1 export from the nucleus and proteasomal degradation in the cytoplasm in keratinocytes^47^. Serine phosphorylation of E2F1 by GSK3β increases E2F1 association with the deubiquitinating enzyme USP11, which removes K63-linked ubiquitin chains thereby preventing E2F1 degradation in the nucleus^48^. Moreover, acetylation of E2F1 by the acetylase p300/CBP-associated factor P/CAF prevents its degradation^49^. Therefore, RCC1 may regulate proteins that participate in the modification of E2F1 to affect its degradation. Another possibility is that (as shown in Supplementary Figure S2) knockdown of RCC1 reduces E7 protein levels. It is known that E7 binds to and degrades pRb, which results in the release of E2F1 from the pRb-E2F complex, and promotes malignant transformation of cervical epithelium^50,51^. Recently, reports have shown that E7 activates E2F1 transcription and increases E2F1 protein levels^52^. Beyond doubt, the reduction in E7 protein levels via RCC1 knockdown induces the downregulation of E2F1. However, how RCC1 affects the steady-state E7 protein levels remains to be studied.

Cell cycle progression is regulated by cyclins and Cdks at several checkpoints, including the G1, G2/M, spindle, and postmitotic G1 checkpoints^53,54^. The G1 checkpoint is the critical point that determines whether cells enter S phase and proliferate. Under normal conditions, DNA damage results in G1 phase arrest, which prevents cells with damaged DNA from replicating and allows cellular repair systems to fix the damaged DNA (Fig. 6e). HPV E7 either releases E2F1 from the pRb-E2F1 complex or activates E2F1 directly. In our model, RCC1 is upregulated in cervical cancer tissues and cell lines by HPV E7. c-Jun transcriptionally upregulates RCC1 via a direct interaction with its promoter. Moreover, RCC1 regulates Cdk1 expression levels via E2F1 to facilitate the G1/S transition. This E7/c-Jun/RCC1/E2F1/Cdk1 pathway leads to the abrogation of the G1 checkpoint in the presence of damaged DNA; this may induce genomic instability, contributing to HPV-induced carcinogenesis.

## Materials and methods

### GEO dataset

Gene microarray expression data were obtained from the publicly available GEO database (GDS3233 [ACCN] (Affymetrix HG-U133_Plus_2.0 array) (www.ncbi.nlm.nih.gov/geo/query/acc.cgi?acc=GSE24080)15.

### Immunohistochemistry

Immunohistochemical detection of RCC1 (1:100, 22142-1-AP, Proteintech) was performed in normal cervix and cervical cancer tissues as previously described^55^. Briefly, paraffin-embedded sections were deparaffinized, rehydrated and treated with 3% H_2_O_2_ for antigen retrieval. After blocking with TBS-T containing 5% fetal bovine serum (FBS) for 1 h, sections were incubated with an anti-RCC1 antibody overnight. The next day, sections were incubated with HRP-labeled secondary antibody for 2–3 h. The peroxidase reaction was initiated using diamino-benzidine tetrachloride (DAB) as a chromogen and nuclei were counterstained with Mayer’s hematoxylin. Immunohistochemistry staining analysis was performed as described^52^. All slides were scored by two independent blinded investigators. Tumor cell proportions were scored as 0 (no positive tumor cells); 1 (<25% positivity); 2 (25–50% positivity); 3 (51–75% positivity); or 4 (>75% positivity). Staining intensity was graded as 0 (none); 1 (weak); 2 (moderate); or 3 (strong). The staining index (SI) score was calculated by multiplying the staining intensity score and by the percentage of positive tumor cells. The staining results were finally recorded as 0, negative (−); ≤4, low expression (+); 5–8, moderate expression (++); and ≥9, high expression (+++). Tumor samples scored (+) to (+++) were considered positive. If the staining interpretation differed between the two investigators, the data for the slide were discarded.

### Cell culture

Spontaneously immortalized human foreskin keratinocytes (NIKS) were cultured on mitomycin C-treated J2-3T3 feeder cells with E medium composed of Ham’s F12 medium and Dulbecco’s modified Eagle medium (DMEM) (3:1) plus 5% FBS. Human telomerase reverse transcriptase-expressing human retinal pigment epithelial cells (RPE1) were maintained in Ham’s F12-DMEM medium (1:1) plus 10% FBS. NIKS cells and RPE1 cells expressing HPV-16 E7 were established using a pBabe retroviral system as described previously^28^. NIKS-derived and RPE1-derived cell lines were maintained in puromycin and used within 15 passages. HeLa and SiHa cells were cultured in DMEM. All cells were cultured in medium with the addition of penicillin and streptomycin at 37 °C with 5% CO_2_.

### Western blot and cell fractionation

Total cellular proteins were extracted with radioimmunoprecipitation assay (RIPA) lysis buffer, and Western blot was performed with specific antibodies against RCC1 (1:500, sc-55559, Santa Cruz; 1:500, 22142-1-AP, Proteintech), E2F1 (1:1000, sc-193, Santa Cruz), Cdk1 (1:1000, 610038, BD Biosciences), Cdk2 (1:1000, sc-6248, Santa Cruz), Cdk4 (1:1000, sc-260, Santa Cruz), SP1 (1:1000, 9389s, Cell Signaling), c-Jun (1:1000, 9165, Cell Signaling), HPV-16 E7 (1:500, sc-6981, Santa Cruz), and Tubulin (1:10 000, T-4026, Sigma). Secondary antibodies included IRDye 800CW goat anti-mouse IgG (1:10 000, 926-32210, Licor) and IRDye 800CW goat anti-rabbit IgG (1:10 000, 926-32211, Licor). Protein bands were detected using an Odyssey infrared imaging system (Li-COR, Lincoln, NE) and quantified using Image J (NIH). E2F1 half-life was measured following cycloheximide (25 μg/mL) treatment and calculated using Half Life Calculator (www.calculator.net).

To obtain cytoplasmic and nuclear proteins, cells were extracted with the ProteoExtract subcellular proteome extraction kit (Calbiochem) according to the manufacturer’s instructions. Briefly, cytoplasmic, soluble, and insoluble nuclear extracts were prepared using the hypotonic buffer, hypertonic buffer, and insoluble buffer, respectively. Equal volumes of cytoplasmic and nuclear extracts were obtained. SP1 and Tubulin were used as loading controls for the nuclear and cytoplasmic fractions, respectively.

### RNA extraction and real-time reverse transcription (RT)-PCR

RNA extraction was carried out using the TRIzol Reagent (Invitrogen) according to the manufacturer’s instructions. cDNA was synthesized using random primers with the PrimeScript™ RT Reagent Kit with gDNA Eraser (Takara) according to manufacturer’s instructions. Amplification of PCR products was quantified using SYBR® Premix Ex Taq™ (Takara) and monitored on a DNA Engine Peltier thermal cycler (Bio-Rad) equipped with a Chromo4 real-time PCR detection system (Bio-Rad). The following cycling conditions were used: initial denaturation at 95 °C for 3 min, followed by 40 cycles of 95 °C for 15 s, 60 °C for 20 s, and 72 °C for 30 s. The PCR primer sequences were as follows:

RCC1 forward, 5′ - GGCTTGGTGCTGACACTAGGC - 3′;

RCC1 reverse, 5′ - CCTCCACTGATGTGTCCCTTC - 3′;

c-Jun forward, 5′ - CCCCAAGATCCTGAAACAGA - 3′;

c-Jun reverse, 5′ - CCGTTGCTGGACTGGATTAT - 3′;

E2F1 forward, 5′ - CATCCCAGGAGGTCACTTCTG - 3′;

E2F1 reverse, 5′ -GACAACAGCGGTTCTTGCTC - 3′;

GAPDH forward, 5′ - GCACCGTCAAGGCTGAGAAC - 3′;

GAPDH reverse, 5′ - TGGTGAAGACGCCAGTGGA - 3′.

Expression levels were assessed in triplicate, normalized to GAPDH levels, and presented as the combined results of three independent biological replicates.

### ChIP assay

Chromatin immunoprecipitation (ChIP) assays were performed using a ChIP assay kit from Millipore (17-10085) according to the manufacturers’ protocol. Immunoprecipitations were performed using anti-c-Jun or control IgG antibodies. PCR was performed with the primers designed to amplify sequences within the human RCC1 gene as follows:

predicted site 1 - forward 5′- CGGCCCAGGACACTTAGTAC - 3′,

reverse 5′ - AGCAATTTGAGGCTTCTTTGG - 3′;

predicted site 2 - forward 5′ -CTGGTCTTGACCTCCAGGG - 3′,

reverse 5′ - CCAGGCTGGGGAAGATTCT - 3′;

predicted site 3 - forward 5′ - TGTCCTGCCCCCCTAGACT - 3′,

reverse 5′ - CCTCAAGAGGGCATGGAGAAG - 3′.

### siRNAs and transfection

Cells were seeded in 6-cm dishes at 3 × 10^4^ cells and cultured in medium without antibiotics for 24 h. Chemically modified Stealth small interfering RNAs (siRNAs) targeting RCC1, c-Jun, Cdk1 and control siRNAs were purchased from GenePharma (GenePharma, Shanghai, China) and transfected into cells using Lipofectamine 2000 (Invitrogen, Life Technologies, CA, USA) according to the manufacturer’s instructions. Cells were transfected with siRNAs at a final concentration of 20 nM. The siRNA sequences were as follows:

sic-Jun, 5′ - GGCACAGCTTAAACAGAAA - 3′;

siRCC1-1, 5′ - TGGAGATGATGGGCAAACA - 3′;

siRCC1-2, 5′ - CAGCAGCCCTCACCGATGA - 3′;

siCdk1-1, 5′ - GATCAACTCTTCAGGATTT - 3′;

siCdk1-2, 5′ - GATGTAGCTTTCTGACAAAAA - 3′.

Twenty-four hours after transfection, cells were treated with DMSO or 10 μg/mL bleomycin and incubated for an additional 36 h. Cells were harvested for protein knockdown analysis by Western blot or for cell cycle analysis by flow cytometry.

### Plasmids and transfection

The pCMV-3Flag-RCC1 plasmid encodes the RCC1 protein and pCMV-3Flag-Cdk1 encodes an N-terminally FLAG-tagged Cdk1 protein in a pCMV-3Tag-1 vector. The pCMV-E2F1 plasmid (#24225, Addgene) encodes human E2F1 protein with a C-terminal HA epitope tag. To construct siRCC1-resistant variants that contain the sequence TGGAAATGATGGGTAAGCA, mutagenesis was performed using the QuikChange site-directed mutagenesis kit and the QuickChange XL site-directed mutagenesis kit (Stratagene). Mutations were confirmed by DNA sequencing. Transient transfections were performed with Lipofectamine 2000 (Invitrogen).

### Flow cytometry

For cell cycle analyses, cultured cells were treated with phosphate-buffered saline (PBS) or bleomycin (Alexis Biochemicals) (10 μg/mL in PBS). Thirty-six hours later, cells were fixed in 70% ethanol, treated with 50 μg/mL RNase A plus 50 μg/mL propidium iodide (PI). The PI-stained cells were analyzed by flow cytometry. Cell cycle analysis was performed using FlowJo software (Becton Dickinson).

For the bromodeoxyuridine (BrdU) labeling experiments, BrdU (final concentration, 20 nM) was added to the medium 2 h before collection of cells. After fixation, cells were permeabilized with 2 N HCl–0.5% Triton X-100, neutralized with 0.1 M sodium tetraborate, stained with monoclonal anti-BrdU (BD Biosciences) followed by treatment with anti-mouse IgG F(ab)2-fluorescein isothiocyanate (FITC) (Sigma), and counterstained with PBS-7-aminoactinomycin D (7-AAD)-RNase A. Immunofluorescent cells were analyzed on a CytoFLEX (Beckman).

### Statistical analysis

All statistical analyses were performed using SPSS 17.0 (SPSS, Chicago, IL, USA). Data are presented as the means and standard deviations (SD). The differences between means were evaluated using Student’s *t* test. *P* values ≤ 0.05 were considered significant.

## Electronic supplementary material

Supplementary-S1

Supplementary-S2

Supplementary figure legends
