# Supplementary figures and images for: Regulator of chromatin condensation 1 abrogates the G1 cell cycle checkpoint via Cdk1 in human papillomavirus E7-expressing epithelium and cervical cancer cells
Source: Cell Death Dis. 2018 May 22;9(6):583. doi: 10.1038/s41419-018-0584-z (PMC5964113; doi:10.1038/s41419-018-0584-z)

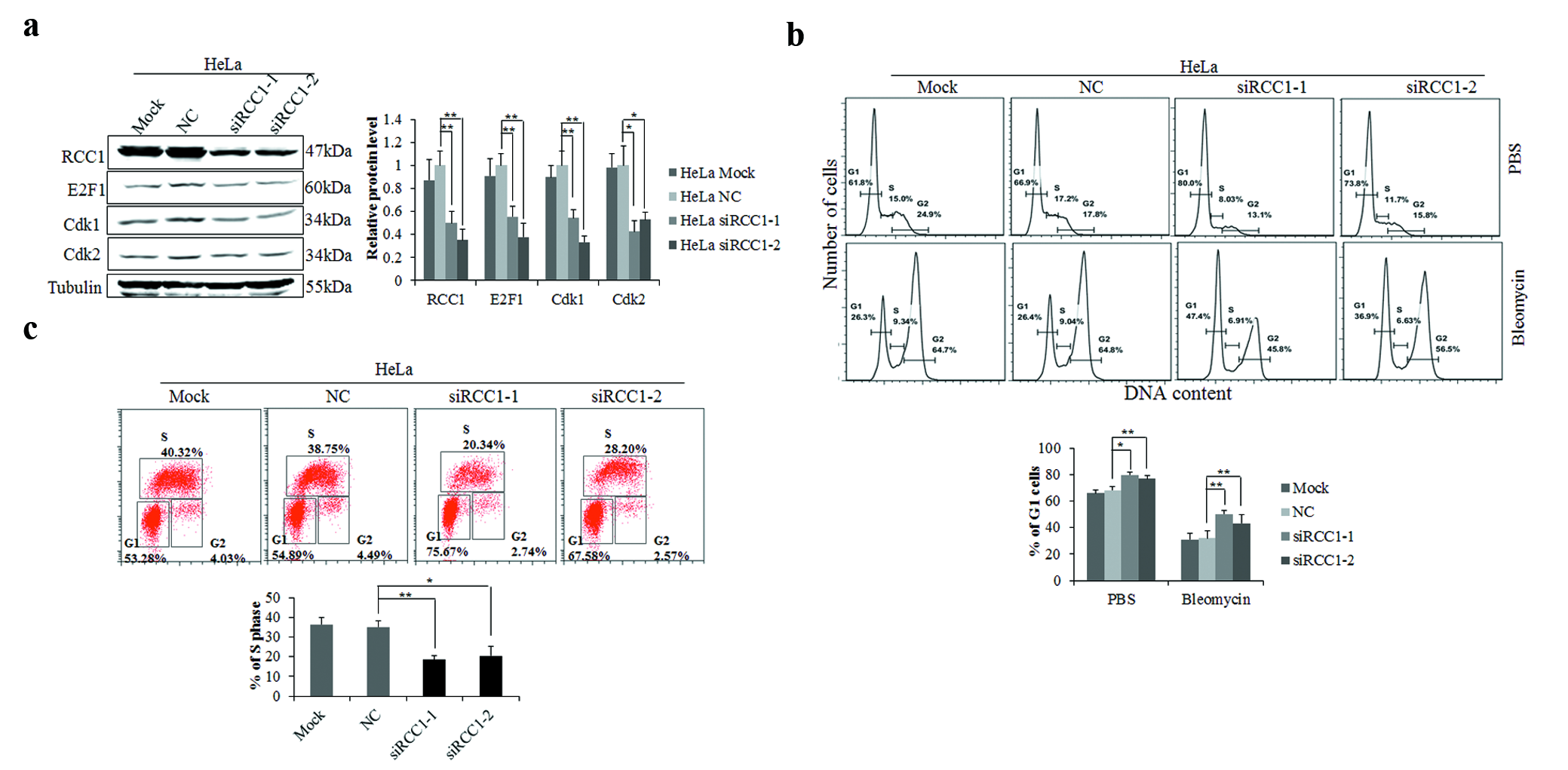

Supplement: Supplementary file 1 — Supplementary-S1 [file 41419_2018_584_MOESM1_ESM.tif]

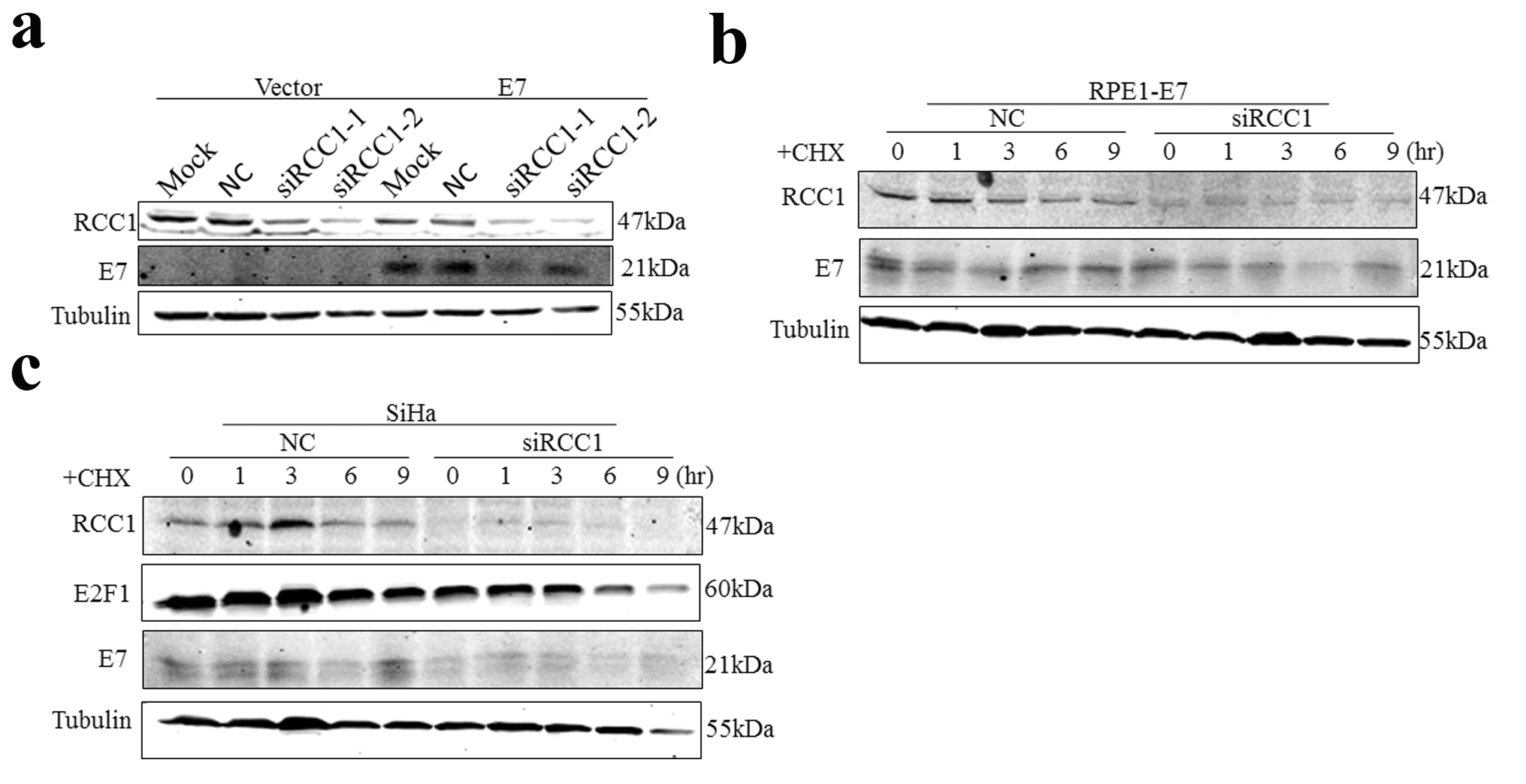

Supplement: Supplementary file 2 — Supplementary-S2 [file 41419_2018_584_MOESM2_ESM.tif]
