# Supplementary material for: Regulator of chromatin condensation 1 abrogates the G1 cell cycle checkpoint via Cdk1 in human papillomavirus E7-expressing epithelium and cervical cancer cells
Source: Cell Death Dis. 2018 May 22;9(6):583. doi: 10.1038/s41419-018-0584-z (PMC5964113; doi:10.1038/s41419-018-0584-z)
Supplement: Supplementary file 3 — Supplementary figure legends [file 41419_2018_584_MOESM3_ESM.docx]

**Supplementary Figure S1** RCC1 knockdown induces G1 arrest in Hela cells. (**a**) Western blot analysis of E2F1, Cdk1 and Cdk2 protein levels in HeLa cells transfected with RCC1 siRNAs. (**b**) Flow cytometry of HeLa cells transfected with siRCC1s for 24 hr and then treated with bleomycin for 36 hr. (**c**) Flow cytometry of HeLa cells transfected with RCC1 siRNAs for 48 hr and then stained with anti-BrdU and 7-AAD. Data representative of 3 independent experiments are shown. **P* < 0.05; ** *P* < 0.01.

**Supplementary Figure S2** RCC1 knockdown decreases E7 protein expression in RPE1-E7 and cervical cancer cells. (**a**) Western blot analysis of RCC1, E7 protein levels in RPE1 cells transfected with RCC1 siRNAs. (**b**) RPE1-E7 cells were incubated with 25 μg/mL CHX and harvested at the indicated times. E7 protein stability was monitored by Western blot. (**c**) SiHa cells were incubated with 25 μg/mL CHX and harvested at the indicated times. The stability of E2F1 and E7 proteins were monitored by Western blot.
